# Long-Term Maintained Response to Selective Internal Radiation Therapy in an Oligometastatic Uveal Melanoma Patient Treated with Concomitant Anti-PD-1 Therapy

**DOI:** 10.3390/life11070692

**Published:** 2021-07-14

**Authors:** Ilaria Proietti, Nevena Skroza, Luca Filippi, Nicoletta Bernardini, Alessandra Mambrin, Ersilia Tolino, Giovanni Rossi, Anna Marchesiello, Federica Marraffa, Salvatore Volpe, Oreste Bagni, Concetta Potenza

**Affiliations:** 1 Dermatology Unit “Daniele Innocenzi”, “A. Fiorini” Hospital, Via Firenze, 1, 04019 Terracina, Italy; nevena.skroza@uniroma1.it (N.S.); n.bernardini@ausl.latina.it (N.B.); a.mambrin@ausl.latina.it (A.M.); e.tolino@ausl.latina.it (E.T.); rossi.1598580@studenti.uniroma1.it (G.R.); anna.marchesiello90@uniroma1.it (A.M.); federica.marraffa@uniroma1.it (F.M.); salvatore.volpe@uniroma1.it (S.V.); concetta.potenza@uniroma1.it (C.P.); 2 Department of Nuclear Medicine, “Santa Maria Goretti” Hospital, Via Antonio Canova, 04100 Latina, Italy; l.filippi@ausl.latina.it (L.F.); oreste.bagni@uniroma1.it (O.B.)

**Keywords:** uveal melanoma, selective internal radiation therapy, immunotherapy, anti-PD1

## Abstract

Uveal melanoma (UM) is a primary neoplasm of the eye arising from the melanocytes residing in the iris, ciliary body or choroid. It is the most frequent intraocular malignancy and often determines metastases at distant sites, with a peculiar tropism for the liver. Metastatic UM has a poor prognosis, as any treatment affects the natural course of this fatal disease. Herein, we report a case of a UM metastatic to the liver in a 54 year-old female patient, initially treated with nivolumab without success. The patient was then scheduled for selective internal radiation therapy (SIRT) while continuing immunotherapy. This combination led to a complete and durable response and the patient is currently free of disease, two years after the diagnosis of the hepatic metastases. The association between SIRT and immunotherapy (IT) has very promising perspectives for metastatic UM, especially considering the disappointing or contradictory results of classic chemotherapies, IT alone and targeted therapies. Furthermore, this combination has been shown to have a good security profile. However, further studies are needed to confirm the efficacy of associating SIRT and IT and to clarify some unsolved problems, such as the timing of administration of these two therapies.

## 1. Introduction

Uveal melanoma (UM) is the most common primary malignant intraocular tumor in adults; it has a very aggressive biologic and clinical behaviour, characterized by a remarkable capacity of hematogenous dissemination and metastasis [1]. 

Though sharing some mutations with the cutaneous melanoma, UM has a quite different molecular landscape, with distinct driver mutations. Among them, the G-protein-α subunits GNAQ or GNA11 mutations have a central role, leading to a constitutive signaling by the RAS/RAF/MEK/ERK (RAS-ERK) pathway; these aberrations increase cell proliferation and tumor growth. Another driver mutation is the inactivation of BAP1, which is found nearly in 50% of all cases. BAP1 encodes for a catalytic subunit of a nuclear ubiquitin carboxy-terminal hydrolase with various substrates, such as BRCA1, thus leading to an enhanced metastatic potential. On the other hand, the mutations found in the splicing factor 3B subunit 1 (SF3B1) and in the eukaryotic translation initiation factor 1A, X-linked (EIF1AX), and amplifications of CNKSR3 are associated with a less aggressive behaviour and improved survival of UM patients; nevertheless, they are rarely found [2].

Concerning therapy, the improvements in primary tumor management through surgery and/or radiotherapy have led to a relatively good response of the primary UM [3]. However, this has not translated into longer survival; in fact, more than 50% of patients with UM end up developing metastases [4]. The most common site of metastasis is the liver (80–90%). To date, there is no consensus on setting the gold standard treatment of metastatic UM [5]. Most systemic treatments derive from the experience in cutaneous melanomas. Classic systemic chemotherapies have shown a high grade of resistance.

Immunotherapy (IT) has dramatically changed the management of cutaneous melanoma. Checkpoint inhibitors are one of the most successful agents used in cutaneous melanoma therapy. Response rates among metastasized patients are in the range of 40–50% for anti-programmed cell death protein 1 (PD-1) therapy. However, the success observed for checkpoint inhibitor treatment of metastasized cutaneous melanoma has not been observed for stage IV UM. Several reasons have been implicated to explain this limited efficacy for the treatment of advanced UM: it is known that liver metastases, even in cutaneous melanoma, seem to respond less well than other types; moreover, UM usually has a low mutational burden, reducing the probability of UM cells to be recognized and attacked by immune cells [6].

On the other hand, promising results have been obtained by using selective internal radiation therapy (SIRT). It consists of the injection of 90 Yttrium (Y)-loaded glass or resin microspheres directly into tumors’ arterial feeder and has proved effective for the treatment of primary and secondary hepatic tumors. The rationale of using SIRT to treat hepatic metastases of UM derives from pathological and clinical considerations: firstly, these metastases are widespread and not eligible to local ablations [7]; secondly, although UM is not a heavily radiosensitive neoplasm, the primary ocular malignancy often responds to high dose RT [8]. 

Recently, based on some evidence showing a synergy between SIRT and IT, a therapeutic approach that combines both of these treatments has emerged with promising results. 

Herein, we report a case of UM metastatic to the liver effectively treated by combining IT and SIRT and a review of the literature about the combination of these two therapies for stage IV UM. 

## 2. Case Presentation

In 2014, a 52 year-old woman was diagnosed with left eye UM. Her medical history was significant for a subclinical hypothyroidism due to a multinodular goiter. She denied having a familiar history of melanoma. 

The patient was submitted to enucleation. A contrast-enhanced computed tomography (ce-CT) was taken in order to stage the neoplasm, and no metastases were detected. Post-operative staging resulted in pT3a N0 M0, stage II A according to the 2009 UICC AJCC TNM staging system. A BRAF mutation testing was taken: the molecular analyses for V600E, V600D and V600K mutation were negative. 

No further treatments were performed, and the patient was submitted to periodic follow-up through clinical and radiological examination. The patient remained free of disease until June 2019, when ce-CT disclosed a gross lesion in the 6th segment of the right hepatic lobe and a smaller lesion in the 3rd hepatic segment, suspected for melanoma metastases, subsequently confirmed by hepatic positron emission computed tomography (PET/CT) scan with 18flurodeoxyglucose (18F FDG), through a digital Biograph Vision 450 PET/CT scanner (Siemens, Germany) The patient started therapy with an anti PD-1 immune checkpoint inhibitor (nivolumab, 480 mg every 4 weeks).

Three months after the start of immunotherapy (September 2019), she underwent a further PET/CT scan with 18F FDG that showed the hepatic metastases mildly increased in FDG uptake (i.e., standardized uptake value, SUV) and volume. After a collegial discussion, also considering the possibility of pseudo-progression due to the start of immunotherapy, the treatment regimen was not changed. Six weeks later, the patient repeated the PET/CT scan and also performed a hepatic MRI, both positive for progressive metastases: the PET/CT scan with 18F FDG showed a single lesion, characterized by highly increased tracer uptake (SUV max 21), in the 6th segment of the right hepatic lobe, evident in the coronal and axial images. Contextually performed hepatic magnetic resonance imaging demonstrated a hepatic lesion in T2 weighted images, with a characteristic hypersignal in the basal T1 sequence, diffusion restriction in diffusion-weighted imaging (DWI) and the apparent diffusion coefficient (ADC) map, contrast enhancement in the arterial phase and no lesion contrast media (CM) concentration in the hepatobiliary phase.

In April 2020, the patient was scheduled for selective radiation internal therapy (SIRT) of the hepatic metastases with ablative intent, without discontinuing immunotherapy, in order to prevent melanoma extra-hepatic spreading. Pre-procedural laboratory tests resulted in gamma-GT 25 U/L, GOT 15 U/L, GPT 16 U/L, bilirubin (total) 10 μmol/L and albumin 40 g/L. The patient’s performance status according to Eastern Cooperative Oncology Group was 0.

In May 2020, before the administration of SIRT, she underwent abdominal angiography and scintigraphy with 99mTc macroaggregates (99mTc MAAs), which demonstrated selective and intense 99mTc MAAs’ accumulation in both hepatic lesions. The abdominal angiography taken before treatment clearly showed the arterial feeder of the lesion. Fused single photon computed tomography (SPECT) and CT images demonstrated an optimal match between the lesion and 99mTc macroaggregates’ location. A sequential right and left lobar approach was planned, with an interval of six weeks between the 1st and 2nd 90Y microspheres’ administration.

As far as it concerned the largest lesion in the 6th segment, the activity to be delivered, determined by the partition model (PM), was 450 MBq, resulting in a dose (i.e., mean dose) to the tumor of 210 Gy. One week after the completion of the pre-procedural examinations, SIRT was performed. SIR spheres were delivered through a micro-catheter, selectively placed into the arterial branch for the 6th hepatic segment, in a slow and fractionated manner, resulting in adequate targeting of the lesion on post-treatment 90Y PET. The treatment was well-tolerated, with neither clinical toxicity nor transitory abnormal values of laboratory tests (i.e., hepatic enzymes’ levels resulted normal during follow-up), and immunotherapy was regularly administered three weeks after 90Y administration, according to the scheduled patient’s protocol. 

**Figure 1:**
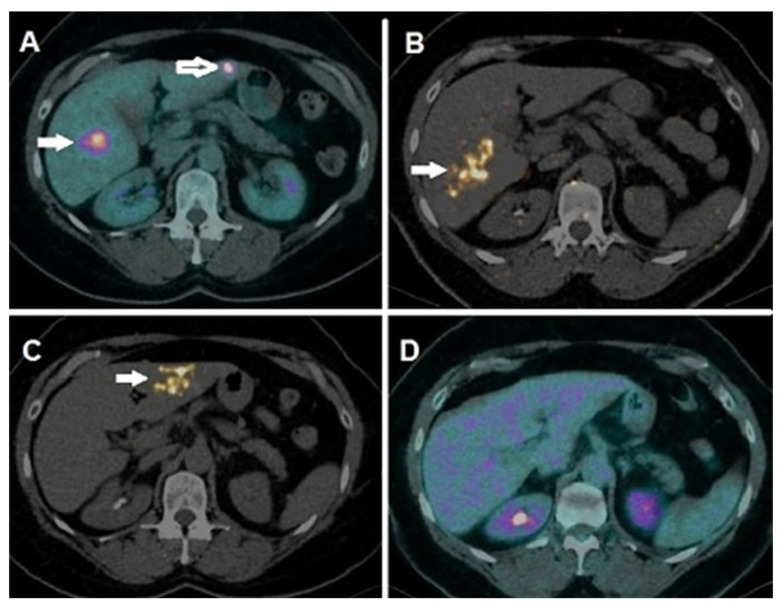
(**A**) Pre-SIRT 18 F-FDG PET/CT demonstrated intense focal tracer uptake in two hepatic lesions located in the 6th (white arrow) and 3rd (white-contoured arrow) segment, respectively. (**B**) PET/CT acquired on 90 Y-positronic photopeak after the 1st SIRT lobar session showed optimal targeting of the right hepatic lobe and metastasis (white arrow). (**C**) PET/CT acquired on 90 Y-positronic photopeak after the 2nd SIRT lobar procedure depicted adequate targeting of the left hepatic lobe’s metastasis (white arrow). (**D**) Four months after SIRT, 18 F-FDG PET/CT showed a complete metabolic response.

In June 2020, she underwent a PET/CT scan (Figure 1) and hepatic MRI that both demonstrated a well-evident hypoactive area in correspondence with the treated tumor, shown by fused coronal and axial slices. Contextually performed hepatic magnetic resonance imaging of the treated lesion (T2 weighted) also showed complete response to 90Y RE, with increased basal T1 signal and diffusion facilitation in the DWI and ADC map, both consistent with necrosis. Neither enhancement was evident in the post-contrast arterial phase nor CM concentration in the lesion, and in the surrounding, hepatic parenchyma exposed to irradiation was detected at the hepatic and biliary phase. These findings were consistent with a complete response to TARE/SIRT. Four months after therapy, the patient was in optimal clinical conditions, still under immunotherapy, free from disease according to clinical and imaging parameters. Subsequently, she was submitted to the 2nd lobar treatment for the radioablation of the lesion located in the 3rd hepatic segment, through a selective arterial catheterization and the administration of 500 MBq of 90Y microspheres, calculated by PM dosimetry, to achieve a dose to the tumor of 200 Gy. The 2nd administration of the 90Y microspheres was well tolerated too. 

In January 2021, the patient was submitted to restaging 18FDG PET/CT (Figure 1) and hepatic MRI: both examinations showed a complete response at the hepatic level; furthermore, PET/CT did not detect any extra-hepatic sites of pathological tracer uptake.

The patient continued therapy with nivolumab 480 mg every 4 weeks; the last follow-up PET/CT was taken in May 2021 and remained negative for abnormal tracer uptakes. 

## 3. Discussion

UM has a remarkable metastasizing potential. UM patients who have undergone successful treatment of the primary tumor still develop metastases. This implies that UM cells begin in the very early phases to spread into the systemic circulation. On this basis, the detection of circulating tumor cells (CTCs) seems to have a promising role in order to intercept, in the early stages, those tumors that have a higher metastatic potential. However, CTCs are often absent in the peripheral blood of patients with primary UM; by contrast, they are easily found in the metastatic disease [9,10,11,12]. In addition to CTCs, the latency time between the primary intraocular tumor and the development of metastases can be explained by the presence of micrometastases. Their existence has been confirmed by both animal models and histopathologic studies on the liver of patients with a history of UM. These micrometastases can remain in a dormant state. An important role in their growth is played by the tumor microenvironment: important genes implicated in the metastatic potential of UM are related to hypoxia and angiogenesis, and there is some evidence that the unblocking of the “angiogenic dormancy” can shift from dormant to fast-growing metastases. Immune evasion is also implicated in the maintenance of micrometastases [13].

Although there is increasing knowledge about the genetics and pathophysiology of UM, the treatment of metastatic disease remains an open challenge. In fact, therapy of UM has a dual reality. If, on one hand, surgery and radiotherapy have allowed for an excellent local control of the disease, on the other, there is no proven standard of care for patients who develop metastases yet. The adaption of the therapies currently used for metastatic cutaneous melanoma has produced disappointing results. 

Classic chemotherapeutic agents used for cutaneous melanoma, such as dacarbazine, temozolomide, cisplatin, treosulfan and fotemustine, even in various combinations, have been investigated; however, to date, their results have been unsatisfactory [14]. 

Ipilimumab, a human monoclonal antibody that blocks the cytotoxic T-lymphocyte-associated antigen 4 (CTLA-4), is approved in the USA and Europe for the treatment of advanced, unresectable melanoma. However, its application in metastatic UM has achieved response rates of 5–10%, with a median overall survival (OS) of 6.0–9.7 months; a significant response has been observed only in a minority of the patients [15]. The phase II GEM-1 trial has shown higher rates of response when enrolling treatment-naïve patients [16]; however, in the phase II DeCOG trial, these results did not translated into a clinical improvement, as the median overall survival remained 6.8 months even if the patients were previously untreated [17]. 

Nivolumab and pembrolizumab, two human monoclonal antibodies targeting the programmed cell death 1 (PD-1) receptor, are approved in the USA and Europe for advanced melanoma. Their results in metastatic UM are yet to be fully described. A phase II trial in patients with metastatic uveal melanoma is currently recruiting (NCT02359851); on the other hand, in a series of seven patients treated with pembrolizumab after progression on ipilimumab, the median PFS was 3 months [18].

The increased acquisitions about the biology of UM have led to various efforts in order to design targeted therapies. The most important molecular target for cutaneous melanoma are BRAF and MEK; however, UM lacks BRAF mutations, because the RAS-ERK pathway is constitutively activated thanks to the GNAQ/GNA11 mutations. Moreover, there is a rationale for treatments that target downstream components of the pathways driving tumor growth, including MEK and protein kinase C (PKC) [2]. 

Selumetinib is an oral, potent and highly selective allosteric MEK1/2 inhibitor, which has been proven to reduce UM cell viability. Over these molecular bases, in a phase II trial, 101 UM patients were enrolled and treated with selumetinib. There were both treatment-naïve or pre-treated patients; the comparison group received chemotherapy (temozolomide or dacarbazine). Median PFS results were significantly higher in the patients treated with selumetinib, but there was not a statistically significant difference when comparing the OS of the two groups. Nevertheless, the assessment of OS was biased because of the crossover of the majority of the patients of the chemotherapy arm to selumetinib [19]. 

In the wake of these results, a phase III trial (SUMIT, NCT01974752) was initiated in order to compare selumetinib in combination with dacarbazine, versus dacarbazine alone, in treatment-naïve UM patients. Even in this trial, the results were disappointing: although response rates were better in the selumetinib arm, the PFS did not differ significantly between the two groups; the OS data were immature at the time of primary analysis [20].

The contradictory results reported by the two above mentioned trials can be related to the different study design: in fact, in the phase II trial43, the study group received a selumetinib monotherapy, whereas in the phase III SUMIT trial, selumetinib was combined with dacarbazine. 

Other clinical trials are currently ongoing.

Trametinib, another MEK1/2 inhibitor, has demonstrated limited clinical activity in 16 heavily pre-treated patients with metastatic UM: the median PFS corresponded to 1.8 months, and the rates of response were null, as no relevant radiologic response was observed [21]. 

Phosphorylated AKT is observed in >50% of uveal melanomas and correlates with its metastasizing capability [22]. Furthermore, selumetinib in combination with the AKT inhibitor MK2206 has shown interesting results in preclinical studies [23]. Thus, the combination of MEK and AKT inhibition offers promising perspectives. On this bases, a phase II trial has been designed to compare, prospectively, the efficacy of trametinib with or without the AKT inhibitor GSK2141795 in patients with metastatic UM (NCT01979523).

There is increasing evidence of a synergy between radiotherapy (RT) and IT. Radiation therapies can provoke a local and systemic immunomodulation and an increasing of the tumor mutation burden; both these effects can cooperate with immune checkpoint inhibitors to allow tumor-reactive T cells to mount a specific response [24]. This is meaningful, especially for UM: in fact, this tumor carries a lower mutation burden compared with cutaneous melanomas, although they share many antigens. This has some pathological and clinical correlates; UM has been shown to have a less represented CD8 infiltrate, with a perilesional rather than intralesional distribution; besides, this lower immunogenicity may also explain the reduced efficacy of systemic anti-PD-1 therapies for UM [25].

SIRT may increase the expression of tumor-associated antigens and the recruitment of tumor infiltrating lymphocytes, thus helping the immune system unleashed by IT to recognize neoplastic cells and express its therapeutic effects, even after the cessation of the local radiation therapy. With this in mind, some studies have highlighted a promising role of the combination between SIRT and IT.

One of them (Levey at al.) [26] managed to prove a clear advantage in terms of overall survival (OS) when combining IT and SIRT: the median OS for those who received immunotherapy within 3 months of undergoing Y 90 was prolonged at 26.0 months, versus 9.5 months for the patients who did not (*p* = 0.014). Median hepatic progression-free survival (PFS) was prolonged in patients treated with Y-90 and con-current immunotherapy at 10.3 months, versus 2.7 months for TARE only (*p* = 0.002).

Other studies did not report a comparison group in order to demonstrate a meaningful advantage in combining IT and SIRT. However, two of them (Ruohoniemi et al., Zheng et al.) [27,28] showed good results in terms of OS and PFS (20 months and 17 months for OS and 7.8 and 15 months for PFS, respectively). Besides, the majority of the patients reported by both studies showed a radiologic response or at least a stable disease, which cannot be considered as a treatment failure.

Two other studies enrolling patients treated by combining SIRT and IT (Arualanda et al., Schelhorn et al.) [29,30] showed a markedly shorter OS in comparison with the others mentioned above. In the first one, SIRT was associated to chemotherapy with cisplatin, and IT was administered when all these treatments failed. In the second one, SIRT was administered in 6 patients previously treated with Sorafenib, a multiple kinase inhibitor; however, only one patient received an immune checkpoint inhibitor (Ipilimumab), which was given after SIRT. Thus, in both these studies, immunotherapy, when administered, was postponed to chemotherapy and other systemic therapies. SIRT was performed before the administration of IT in both studies. These factors may explain the worse outcome of the patients. 

Concerning the timing of immunotherapy and SIRT, there are no clear indications, and various regimens have been used. Ipilimumab, Pembrolizumab and Nivolumab have been used in variable schedules, doses, timing and sequencing, but generally before SIRT at the time of the diagnosis of hepatic metastases. The literature is not unanimous concerning the best sequencing of these treatments. However, a systematic review highlighted that survival tended to be longer for concurrent compared to non-concurrent treatments, although without statistical significance [31].

On the other hand, there has been more consensus about the fundamental role of the PET/CT for these patients. PET/CT surely has a prognostic significance in evaluating the response: a complete response is relatively rare. However, a partial response and stable disease are more frequent. PET/CT has a clear role, even in prediction of therapeutic success before SIRT. Metabolic tumor volume and total glycolytic activity are predictive functional tumor parameters, which may facilitate patient selection and risk stratification, and volumetric dose calculations showed a statistically significant association with the metabolic tumor response. [32,33]. 

Concerning security, the association SIRT-IT has been proven to be safe. In this regard, it has to be underlined that SIRT cannot be considered a standard adjuvant treatment after PD-1 inhibition, but rather, a potentially useful combination of a loco-regional and systemic therapy, whose optimal sequence still has yet to be fully addressed.

One study (Leppelman et al.) [34] clearly showed that the rate of adverse effects is similar when comparing IT alone and IT combined with SIRT. Considering all the studies selected, only one (Ruohoniemi et al.) reported G4 adverse effects (two cases: gastrointestinal bleeding and hepatic abscess). However, in all the studies, the majority of the reported adverse events were G1 and G2; these side-effects did not lead to suspension of IT.

It has to be highlighted that, aside from GI adverse effects and transitory fatigue, the most relevant complication of SIRT is represented by radioembolization-induced liver disease (REILD), with a reported incidence of less 1% in the largest cohorts [35]. REILD has firstly been described by Sangro et al. and is characterized by hepatic sinusoid obstructive syndrome, ascites, weight gain, and liver failure, with a drastic downhill course [36]. A meta-analysis performed by Braat et al. [37] evaluated the impact of REILD in an overall number of 26 clinical studies: among them, 15 reported the occurrence of REILD without a precise clinical description, while in the remaining 11, a clinical syndrome was described with ascites, hyperbilirubinemia and increased weight. Several risk factors for REILD have been identified, such as previous chemotherapy, previous intra-arterial treatments or repeated SIRT sessions [38].

As far as it concerns the case we have described, no REILD-associated risk-factors were present. Furthermore, since our patient presented metastases located in both hepatic lobes, we decided to perform a sequential lobar approach (i.e., a 1st ^90^Y-administration to the right lobe, followed, after an interval of 4–6 weeks, by a 2nd ^90^Y-administration to the left one). Sequential SIRT has been found to be associated with a lower hepatic toxicity as compared to the simultaneous administration of ^90^Y-microspheres to both hepatic lobes in a single session, the so-called “whole liver” approach [39]. The aforementioned issue might represent an explanation of the optimal tolerance, without clinical and laboratory signs of toxicity, demonstrated by our patient to SIRT.

A further consideration has to be made regarding SIRT repeatability in case of a patient’s relapse after a first administration of ^90^Y-microspheres. In such a case, a preliminary report by Badar et al. [40] investigated the clinical outcome of 26 subjects who received repeated SIRTs to treat recurrent or residual primary disease in similar hepatic arterial lobes or segments: A Kaplan–Meier analysis did not show a significant difference in overall survival between subjects receiving a single ^90^Y-administration and those who were treated with repeated SIRT-sessions. However, due to the limited scientific data available on this topic, it should be kept in mind that the choice of repeated SIRT sessions in relapsing hepatic tumors has to be evaluated on a case-by-case basis, as well as through accurate dosimetric calculations of the radiation burden delivered to the tumor and healthy parenchyma [41].

## 4. Conclusions

Typically, when a melanoma patient does not respond to anti-PD-1 checkpoint inhibitors, the treatment regimen has to be modified, considering a switch to molecules with different mechanisms, such as anti-CTLA-4 (ipilimumab). Our patient showed a complete response at the hepatic level to SIRT and concomitant anti-PD-1 therapy after a progression phase and was free of disease after a 14 months follow-up from the SIRT administration. A combination of both approaches resulted in a long-term maintained remission and could be considered as a valid therapeutic alternative in order to enhance immunotherapy effectiveness, avoid a pharmacological switch, and achieve a better prognosis.
